# The Student in Medicine1Address delivered at the opening of the Medical Department of the University of Buffalo, September 25, 1899. Reported stenographically for the Journal.

**Published:** 1899-11

**Authors:** William H. Heath

**Affiliations:** Buffalo, N. Y., Professor of genito-urinary diseases, University of Buffalo


### BUFFALO MEDICAL JOURNAL.

VOL. XXXIX.—LV. NOVEMBER, 1899. NO. 4.

### Original Communications.

### THE STUDENT IN MEDICINE.[1]

BY WILLIAM H. HEATH, M. D., Buffalo, N. Y.,

Professor of genito-urinary diseases, University of Buffalo.

AFTER accepting the duty and honor of opening the college session with the customary address, in considering the matter the sorry thought occurs to me that there is nothing very new to say, nothing that has not probably been said before and much better.

There is the usual consideration of the student, the college and, finally, the panegyric of medicine. Some question the value or necessity of the introductory address, considering it more pertinent for each chair to devote a portion of its first lecture to a presentation of the subject at large, its scope, methods of study, and the like, very much on the order of a preface of a book, a part which is too often unread.

Abernethy once said, on beholding the large class at his first lecture, "My God, what is to become of you all?" The same thought occurs to me, but modified by the changes time has brought. 'It is not now "what is to become of you all," but "what may not become of you." Medicine today is in a transitional state more than ever before. New light is being thrown upon old phenomena, new ones discovered, and from the most unexpected quarters. Empiricism is disappearing and the art is fairly resting upon a scientific basis.

Most of the just criticism of our profession, which in the past was in no small measure due to ourselves, has been largely corrected. The deficiency of preliminary education and faulty medical training

---

1. Address delivered at the opening of the Medical Department of the University of Buffalo, September 25, 1899. Reported stenographically for the JOURNAL.

have been corrected and a more careful self scrutiny of those who enter the profession has occurred. With this change and transition and advance and with the possibilities of the future you are concerned. You are all the architects of your own fortune. Work is to be done in which you can and will participate and which may lead you to the top, which is broader, though higher than ever before. It was said years ago that every French soldier carried a field marshal's baton in his knapsack. You may not be field marshals in medicine, but the same possibilities may carry you to fame and fortune. Success demands very much, the best there is in you and from the beginning. There is no royal road to, nor trusts in, learning, and though the possibilities are greater, the standard is higher, competition keener, the problems more subtle, but the rewards are proportionate. If you wish to succeed you must take the turnpike, which is hard, and trudge along the way those who have gone before you and succeeded have traveled.

It is hoped that you who are about entering this field have been influenced by proper motives, due consideration and not by the superficial appearance of attractiveness, such as independence, the appearance of authority, or ease. Either from want of knowledge of themselves, or the requirements of medicine, or proper lack of advice, unquestionably more enter this profession that are ill adapted to it than any other. If the details appear dry, the study disagreeable, parts distasteful, you are not intended for a successful doctor and stay your steps before advancing.

It would appear pertinent therefore to here refer to some of the features which observation and experience have determined to be of value upon entering this career.

No one should consider the serious study of medicine who is superficial, trivial or weak. Too many have done so. It is a dignified calling demanding dignity. Serious because it brings you into the heart of the family, in touch with suffering, and into the shadow of death ; even more than the priesthood, or any profession.

No one should consider the study of medicine who has not had a suitable preliminary education. As well attempt to build a palace of stone on a foundation of mud and straw. It is necessary in the acquirement of the principles of medicine and further in life to permit the growth of other culture. The public and patients expect the doctor to be more than a medicine man, more than a detective, to detect pathological specimens, more than an accountant to strike off a balance, and diagnosticate, from one's statement, his condition.

A natural taste. This is a difficult quality to describe, though we can recognise it. It stands, however, for future enthusiasm, success and the ability to obtain and give the greatest benefits, make the greatest sacrifice, and to find in his art the essence, the something that lightens its labor and tempers the way.

Sir Benjamin Brodie says that he was generally supposed to have a particular liking for his profession, but that it was not so, and that his experience led him to have no faith in those special callings to certain ways of life which some young men are supposed to have and that those who succeed best are those who persevere, as a matter of duty or because they have nothing better to do, until they acquire knowledge and, finally, attain some degree of credit, after which everything is changed and every day they become more interested in their work.

A healthy, strong constitution. It is a trying vocation, as it often says, eat and sleep when you can, take little recreation and very much hard work. Besides, very often a large physique implies a big brain and bigger heart, qualities which are not a drug on the market.

Adaptability, particularly when accompanied by uprightness of purpose, because it engenders the right spirit, and foreshadows a philosophical mind, which may enable you to take the small things of life and walk grandly among them.

A cheerful temperament. You should be an optimist, have plenty of buoyancy and hopeful temperament, because the gloomy visaged, pesimistic, complaining or depressed manner cannot be depended upon to impart hope and buoyancy or dispel depression so often felt by the sick, as well as observed in the sick room.

A level head and judicious mind, because while advantageous in all things it is particularly so in medicine, so often the stalking ground of fads and isms, and because a scratch too much of a pencil, or cut too deep with a knife, may poison a child or bring one nearer death's door than the principles of medicine intended.

That he should be many-sided, because it goes without saying, you treat people as well as their ailments and will come in contact with all sorts, and under all kinds of circumstances. Because it will enable you to study human nature and cultivate the social as well as the studious side of life. For you to abstain from society and cultivate only a student's life, or be a bookworm is a mistake. Bookworms are rarely good doctors. A knowledge of human nature will enable you to be a boon to the sick, and be to yourself a protection from many dangers which only come to a doctor's life; besides it enables you to have control of your patient and maintain it; because its usefulness has no limitations in all affairs of life.

To be resourceful and quick, because you must learn much and have your knowledge with you and adapt it to very many opposite and different wants. In this respect differing very much from the lawyer and men of the cloth. Because it is part of the art to be able "to cut your cloth to fit the man" and in study to know what to digest and what to leave out.

Character, because student life, and the practice of medicine, is full of temptations and often surrounds you with the influences of the most varied and at times of the most dangerous nature. While at college the absence of the school restraint, temptations of youths in large cities, rich idle fellow-students, make it imperative that from the beginning of your career you should be strong in yourself, cultivating the good and eschewing the evil, that the habit be so formed that when in practice the close sacred relations given you by your patients, the position your profession gives you in the community, be justified, and because without it your virtues even may be made vices.

Dress and manner both much proclaim the man. Let your dress be as any gentleman's should be, and do not attire yourself to emphasise your calling, nor should your manner be artificial, nor a veneer. Do not forget that in these times rudeness will not pass for brightness, nor brusqueness for genius. Your appearance and manner should then be temperate, as no one is so open to criticism as a physician, his appearance and behavior. They are sometimes the means of a first start in practice.

Tact. A requisite to success in this as in no other vocation. It is acquired by observation, made easv with good breeding and a level head. The whims of individuals, intensified by sickness, the peculiar and trying environments made by patients and their friends or relatives, make it a most important quality. It aids in getting a practice, keeping it and warding off much unpleasantness. It should be cultivated, but not to the extent as to be hypocrisy, which would only hold you up to ridicule, and deserved remark. It should be varied as the conditions which it is intended for.

Hoping you already possess or will cultivate most of these qualifications, and that, not like the tramp, you will not feel "that advice is but the one thing in the world you have a sufficiency of," let me refer further to some of the features which you will be confronted with later on in your medical life.

The proper study of mankind is man. He it is we are concerned with. He is the material upon which we are to exercise our craft. Therefore, there is nothing which concerns him, which a medical man will not be the better for knowing. Each case that we come across is a case of a disease and a man. The disease element is provided for by the curriculum; the man or personal element can only be learned by experience. Therefore a medical man should be a man of the world. Do not let the sharp interest in medicine prevent you from widening your general knowledge in every way. You should keep up general reading, take an interest in all that is going on in the world, and lose no opportunity to add to your general knowledge. In your everyday work you will be brought into contact with every player on the stage of life. And if you would enter into the spirit of its action, you yourself must be a man of many parts. These remarks may seem premature, but it is just when the student's mind first becomes engrossed with many subjects, as in the medical college, that there is the greatest danger of general knowledge and the love of culture being neglected or lost. Again, though it is true that the earnest worker finds the most happiness in his own work, it is none the less true that much happiness, profit and relaxation can be acquired from outside interests. Hard work with no hobby is like a boiler without a safety valve. It is liable to burst. Some men are so engrossed with work that they cannot take a holiday, or even enjoy one, and should they in due time be able to retire, instead of spending their declining years in well-earned leisure, they break up and die for the want of really something to do.

Someone once said that "man spends one-half his life learning how to make himself miserable the other half."

The course of study resolves itself into two periods: first, that concerned in the study of the structure and functions of the healthy human body, and such subjects and sciences as elucidate them; and, second, in the study of disease of all aspects.

The importance of thoroughness and method in your earlier years cannot be exaggerated. They possess a double value. Not only are they indispensable to the student, but in the profession they afford an invaluable training of powers of observation, and a means of acquiring a scientific habit of mind. They have, therefore, both a technical and a general educational value. Of your early studies anatomy and physiology demand the most thorough and painstaking work. I speak of these because they are the foundation of medicine and what applies to them applies to all. Frequently the student will find himself confronted with matters in them for which he cannot see the remotest possibility of any particular application. None the less on this account must he endeavor to master them, for who, indeed, can pick out the smallest fact in these studies which is necessarily devoid of all practical importance or may become so in anatomy.

Above all, he should not neglect practical work in the dissecting room. The thorough knowledge of anatomy, which will prove of inestimable value to him, cannot be rightly learned by reading, which in comparison is a sham. He may know every artery and vein, be able to draw the most difficult region on the blackboard with his eyes shut, yet fail to recognise even the most important structures he has read of when he meets them in the right, but unusual to him, place, the body. He may (and we are familiar with him) pack and cram himself full with extracts and formulæ in a few weeks to try to represent a year's work, he may pass his examinations, but it will disappear as quickly as it came and need not be mourned, for from a practical view it is valueless. Too strong a plea, therefore, cannot be made for individual and practical work, and particularly in anatomy. The medical curriculum may appear crowded, perhaps over-crowded and will look to you very formidable and almost discouraging. If discouraged, think of the fable of the dial and the pendulum who were discussing matters in general, when the pendulum stopped swinging, appalled at the work before him for a year, said "it is not the stroke that I complain of, I could manage it without much fatigue, but think of the millions I will have swung in the year." "Very good," replied the dial, "but recollect that, though you are thinking of the million, you are only required to perform but one swing at a time, and that a moment will be given you to swing in." No matter how large the curriculum, you will only take it piecemeal.

A grave error into which the medical student often falls, and is likely to, is the getting of only enough knowledge to pass an examination successfully. Examinations are a necessary evil, for it is only by their means it is possible to learn, though imperfectly, that a student has acquired a sufficient degree of learning to entitle him to official permission to practice. Some such qualifications must be had; the public which place their lives in our hands, the state which entrusts us with this responsibility, and the profession demand it. But it is disastrous for you to regard this test as the final object of study, and yet how often is not the mistake made.

The result is the same, no thoroughness or method, the work paroxysmal and punctuated by long intervals of more or less quiescence. In these circumstances the one idea is to induce the brain to assimilate for the time being the necessary store of facts which must be his, condensed and arranged, merely to be brought out in the time of need. It is a travesty of knowledge to call this education. The knowledge thus obtained lacks, among other things, perspective. Each fact stands out of all relation to its surroundings. Such knowledge cannot be called upon for practical application. Let me urge you, therefore, to look as much to the quality as to the quantity of your work. Do not crave too much specially prepared knowledge, or you will not only lose the power of teaching yourself but of applying that which you do know. Remember that knowledge which you can't use is like money you can't spend.

You may be tempted to "read some at home." This is a mistake. The great majority of students can grasp more by listening for an hour to a capable lecturer than in spending similar time on his text-books. It is proverbially easier to convey and express ideas in speech, where the individuality of teachers is a help, with his illustration and criticism in impressing the facts upon you. Lecturers can emphasise and avoid loss of point by repetition, which would not be permitted in a book, and are able to quote similes and illustrations which would appear trivial reduced in black and white.

Often students lose much of the chief value of the lecture by misdirected energy in note-taking. It is foolish to try and take down nearly every word and then spend the evenings in writing them out. It amounts to giving much labor in making what is practically an unrevised and imperfect book. The weak point is that he gains nothing during the lecture, except a lot of written material which when utilised has lost the personal stamp of the teacher. Proper note-taking should only include the headings of the arguments, or the facts for future reference ; in this way he goes along with the teacher, and has his attention fixed, while his assimilation is not interfered with.

Students often meddle with affairs that do not concern them. Nothing is gained and much lost by a first-year student going where he cannot understand, to clinics. That desire is not much higher than the curiosity which attracts one to a street organ or an ambulance. Without understanding, the sights are meaningless tragedies, unsoftened by the real interest they will have after he comes to study, and not to gaze at them.

It may be that the matter of student's recreations, which are not provided for in the curriculum, hardly come within the scope of the occasion like this. Whatever form is adopted, let it be indulged in with some sort of method. One student thinks of nothing but his work, day in and day out, week in and week out, Saturday afternoon and Sunday. It is work, work and read medicine, no relaxation. On the other hand, there is the student who thinks of nothing but play or athletics and getting a place on the football team, perhaps thinking of combining business with pleasure in affording him an opportunity to study the first aid to the injured or become acquainted with fractures. Cultivate the habit, if you can, of throwing off all work at the time for rest, and not relegate the hard study problems to hours known as "spare moments."

In a medical school the social life of a university is absent; a man if he chooses, may never really know those he meets daily. This is undesirable. The man who keeps to himself is denying himself one of the most valuable means of preparing for his part in life.

We are more or less roughly cut stones, few diamonds, and are much improved by proper association with others of like kind.

Make friends. Emerson says, "our chief want in life is some one who can make us do what we can." This is the service of a friend. Therefore, for general and individual advantage, do all you can to promote friendship. No doubt many a medical student has gone to the wall for the want of a real friend to help him with his troubles, anxieties and fears.

To those who look forward to hospital work, much of what has been said also applies and the habit of method and thoroughness which he has cultivated, will yet prove of great value. In the hospital and dispensary, the two great faculties the student of medicine should cultivate are, first, power of accurate observation, and, second, the drawing of conclusions from facts; faculties not different from those aimed at in any form of education, general or special.

It has been said "that scientific training is not the imparting to others the results of someone's training, but in developing in each one a mind capable of making its own observations, its own conclusions." It is not so much how much you know, but how much you are able to find out for yourself. No period of your medical career will compare as training in the art of observation, as the time spent in clinics, dispensaries and hospitals. The cases you will study there will, if rightly studied and observed, lay the best foundation for your future success. One case well studied is worth a dozen slipped over. Each case should be followed through with the utmost care and no detail, however trivial it may appear, should be passed undervalued. Each case thoroughly investigated and recorded will be indelibly fixed on the memory and serve as a standard for after comparison.

The taking of histories is probably the best of the training. It will often seem that the minuteness of details and elaboration of written reports are a waste of time. There is no greater mistake than this. The training thus obtained in the observation of symptoms and physical signs is invaluable; even if it shows your patient is sound, time spent is not wasted. You will often be asked if a patient is in a state of disease or health, and to state the facts you must leave no stone unturned. There are more mistakes in diagnosis from incomplete examination than from false deduction. The latter is excusable, though serious, the former less serious, but perchance inexcusable. In this way only can the living picture of the disease be best studied and particularly when followed by systematic reading. One whose knowledge of disease comes from books is sadly off, for it is proverbial that many of those with the greatest book stock of learning are by no means those who best know how to apply it. So, their opinion in sickness is vastly inferior to that of the man who with less quantity of knowledge has cultivated observation with study.

Remember also that as no two persons called healthy are exactly alike, neither are any two diseased ones precisely similar. Every case presents something new and will differ more or less from the standard type. So much more the necessity of cultivating and exercising observation and judgment. Again, it is often the case that the most prominent symptom, which is most complained of, is less in importance than others more obscure. For this reason also you will see the advantage of trained observation. The ideal clinical teacher is he who possesses this art in the highest degree, and can also analyze for the student and those less gifted, the steps in the process by which he arrives at his conclusions. It is useless for you, and don't try, to attempt brilliancy in such matters, for jumping at conclusions is not only useless, but creates a spirit of carelessness. It is enough for you to study his process and adapt it for your own. Only in this way will you come to have faith in your own opinion, a sound basis for conclusions, and confidence in your own opinion, which, if you demonstrate, others certainly will not have. Carlyle says, "what we call genius is that which enables a man to express what all men are not far from saying and are willing to say." We should all be more like geniuses if we had the courage of our convictions, and had the courage to say it.

I am also reminded of the necessity of broad-mindedness in your medical study. Do not allow any special aptitude for a particular branch to cramp you in other directions. For the present your aim should be to obtain such a general sound knowledge of principles and practice of the science and art as the few years will allow you to. This is an age of specialism, and before you know the names of the bones almost your friends and fellow students will ask what specialty you are going to take up. Tell them general medicine. Specialty is necessary on account of the enormous scope of the science of medicine, and men must be found to limit their efforts, and in so doing acquire a degree of skill impossible to the general practitioner whose sphere knows no such limits. Until you have shown yourself possessed of general knowledge and experience you have no right to be a specialist. A specialist should know everything known about something, and something about everything else. He too often knows something about something and nothing about anything else. Cavities have a general charm for him and gradually his view of life and disease is limited and grows as narrow as the orifice to the particular cavity. He becomes like the frog in the caves of Kentucky, with rudimentary eyes, but no sight; their eyes have long disappeared for want of use.

This department habit of mind is always narrowing and tends to foster sometimes ridiculous extremes and impair the power of forming true conception of the case.

As an illustration, the *Twentieth Century Practice of Medicine* has just been published and is written by as many doctors. The nose, for instance, is handed to an eminent authority in London, but, lest the weight of it should be detracting, the nasal pharynx they sent to France, while the larynx is brought over to North America, leaving the trachea and bronchi behind, which, perhaps, being considered commonplace, has been sent to Scotland, while the remainder of the respiratory system, the lungs, have been sent to South America, handicapped, however, that they should not be considered in relation to contagious diseases; and, finally, the accessory sinuses and the bacteriological features are further distributed, one to San Francisco and the other to a Japanese, I believe, sojourning in Germany.

After all this, I fear you will think medicine is only laborious; that it is a vocation without its pleasures. Not if pleasure consists in anticipation, realisation and reflection. Medicine is full of them. Like the explorer and sportsman, the difficulties of the struggles and hardships are soon crowded down by the memories of successes, and the pleasure of achievement. Especially is this the case if medicine is approached from the scientific standpoint.

Remember, the question for you to decide and to determine is not what sort of a career is medicine, but what sort of a man are you for medicine.

